# Single‐Crystalline Rhodium Nanosheets with Atomic Thickness

**DOI:** 10.1002/advs.201500100

**Published:** 2015-05-08

**Authors:** Li Zhao, Chaofa Xu, Haifeng Su, Jinghong Liang, Shuichao Lin, Lin Gu, Xingli Wang, Mei Chen, Nanfeng Zheng

**Affiliations:** ^1^ State Key Laboratory for Physical Chemistry of Solid SurfacesCollaborative Innovation Center of Chemistry for Energy MaterialsEngineering Research Center for Nano‐PreparationTechnology of Fujian Province, and National Engineering Laboratory for Green Chemical Productions of Alcohols‐Ethers‐EstersCollege of Chemistry and Chemical Engineering DepartmentXiamen UniversityXiamen361005China; ^2^ Institute of PhysicsChinese Academy of SciencesBeijing100190China

**Keywords:** anisotropic growth, carbon monoxide, 2D nanostructures, nanosheets, rhodium

## Abstract

**CO confinement strategy for ultrathin Rh nanosheets**: CO is introduced as a confining agent to regulate the anisotropic growth of unique 2D structure. The single‐crystalline Rh nanosheets have a thickness of three to five atomic layers and tunable edge length ranging from 500 to 1300 nm. By understanding the formation mechanism, surface‐clean Rh nanosheets are also prepared and display better catalytic performance that their surfactant‐capped nanosheets.

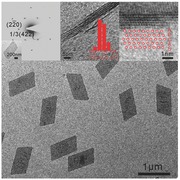

Since the booming development of graphene,^1^ freestanding 2D nanosheets with atomic thickness have drawn extensive attention from scientists due to their unique electronic structure and physical properties compared with their bulk counterparts.^2^, ^3^, ^4^, ^5^, ^6^ The atomically thick 2D nanomaterials show great promises in designing practical applications such as flexible and transparent electronic devices,^7^, ^8^ high‐performance in‐plane supercapacitor electrodes,^9^, ^10^, ^11^ and high‐efficiency catalysts.^12^, ^13^, ^14^ Considering the potential of outperforming their bulk counterparts, great efforts have been made to fabricate atomically thick 2D nanosheets. For graphene and related materials with weak van der Waals forces between layers, they are easily exfoliated into 2D nanosheets through the peeling‐off process.^15^, ^16^ For nonlayer structured materials, it is a real challenge to fabricate their corresponding freestanding 2D nanosheets due to the lack of driving force for anisotropic growth of 2D nanostructure. Most success has been limited to metal oxides^17^, ^18^ and transitional metal chalcogenides.^19^, ^20^, ^21^ 2D metal nanosheets with atomic thickness are rarely reported. Some examples are nanosheets of Pd,^12^, ^22^, ^23^ Au,^24^ Rh,^14^, ^25^ Ru,^26^ PdAg,^27^, ^28^ PdAu,^29^ AuAg,^30^ and PtCu.^31^ This phenomenon is due to metal atoms tend to form 3D closed‐packed structure and lack a strong confining force to regulate the anisotropic growth of the sheet‐like structure. Our group recently developed a CO‐confined growth strategy to prepare freestanding ultrathin hexagonal Pd nanosheets with a thickness of only 1.8 nm. The preferential strong adsorption of CO on the Pd (111) surface was essential to prohibit the isotropic growth and thus result in the ultrathin 2D structure.^12^ Zhang and co‐workers^24^ reported in situ synthesis of gold nanosheets on graphene oxide with thickness about 2.4 nm. The confining force could come from directing template that promotes the anisotropic construction of 2D structure. Formaldehyde‐assisted synthetic strategies have been successfully developed for the preparation of 2D nanosheets of Ru,^26^ Rh,^14^, ^25^ and PtCu.^31^ Similar to metal nanoporous materials,^32^, ^33^, ^34^, ^35^ 2D metal nanosheets exhibit high surface areas and thus high performance in catalysis. Many 2D metal nanosheets also display unit optical and electronic properties.^12^, ^23^, ^27^, ^28^, ^29^, ^30^

The use of certain surfactants, additives, or reducing agents has been well documented in the literature as a crucial to the controlled synthesis of metal nanosheets. However, it still remains much challenging to deeply understand how those parameters induce the anisotropic growth of 2D metal nanosheets. We now demonstrate in this work the synthesis of freestanding single‐crystalline rhodium nanosheets with thickness of three to five atomic layers and tunable diameter (hundreds of nanometers to micrometers). Systematic studies revealed the critical role of CO in regulating the formation of formation of Rh nanosheets. Small rhodium carbonyl clusters were formed at the early state of the synthesis and identified as important intermediates to induce the formation of Rh nanosheets. By understanding the crucial role of CO in the synthesis, both polymer‐capped Rh nanosheets and surface‐clean freestanding Rh nanosheets were successfully prepared, providing us with a great opportunity to figure out the effect of surface coating on their catalytic performance of ultrathin metal nanosheets.

In a typical synthesis of Rh nanosheets, rhodium (II) acetate and poly(vinylpyrrolidone) (PVP) were dissolved in *N,N*‐dimethylformamide (DMF), resulting in a homogenous blue solution in a glass vessel. After being charged with CO to 1.0 atm, the vessel was heated from room temperature to 150 °C with a heating rate of 1 °C min^−1^ and maintained at 150 °C for 3.0 h under stirring. As the reaction proceeded, the color of the reaction mixture changed from blue to brownish‐yellow and finally brownish‐black. After the mixture was cooled down to room temperature, the resulting products were precipitated by acetone, separated through centrifugation, and washed several times with mixture of ethanol and acetone.

The obtained Rh nanosheets were structurally characterized by transmission electron microscopy (TEM). As illustrated in **Figure** **1**, the rhodium nanosheets prepared under 1.0 atm of CO had a uniform morphology of parallelogram (Figure 1a). The long‐edge length of the parallelogram is 880 nm, while the short one is 450 nm. The lattice fridge with an interplanar spacing of 2.33 Å, observed in the high‐resolution HAADF‐STEM image, also matched well with 1/3(422) fringes of face‐centered cubic (fcc) Rh. In the X‐ray diffraction (XRD) pattern of the collected powder sample of Rh nanosheets (Figure S1, Supporting Information), three major diffraction peaks at 2*θ* = 40.8, 47.6, 69.6° ascribed to the diffractions from (111), (200), (220) planes of fcc Rh (JCPDS NO.01‐1213) were clearly observed. The selected area electron diffraction (SAED) pattern of a single rhodium nanosheet (Figure 1c), with its basal plane perpendicular to the electron beam, matched well with the pattern obtained along the [111] axis for fcc Rh as well. These results clearly indicate that the as‐prepared rhodium nanosheets were of fcc structure with (111) as basal planes. 1/3{422} reflections are normally forbidden in bulk fcc metal. The appearance of 1/3{422} reflections in the obtained Rh nanosheets could result from the ultrathin nature of the Rh nanosheets or the occurrence of stacking faults that are parallel to the basal (111) planes.^12^

**Figure 1:**
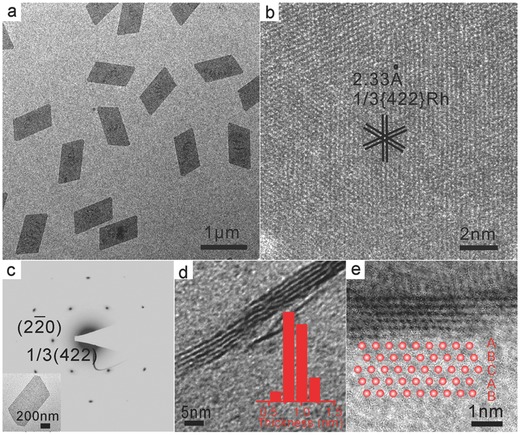
Characterizations of Rh nanosheets. a) TEM image of Rh nanosheets with a long‐edge length of 880 nm. b) High‐resolution HAADF‐STEM image projected along the [111] axis. c) SAED patterns of a single Rh nanosheet (shown in the inset). d) TEM image of the cross‐sectional Rh nanosheets obtained by sectioning using a microtome. The inset shows the thickness distribution of the Rh nanosheets. e) High‐resolution bright‐field image of the cross‐section of a single rhodium nanosheet showing ABCAB stacking sequence along the [111] axis as shown in the inset.

Due to their ultrathin feature, the obtained Rh nanosheets tend to lie flat on the TEM grid, making the direct measurement of the thickness even harder. To have a clear picture of their thickness, Rh nanosheets were embedded in epoxy resin, and thin sections of about 50 nm in thickness were obtained by microtoming the resin sample. The high‐resolution transmission electron microscopy (HRTEM) images of the cross‐sections of the Rh nanosheets were obtained to analyze their exact thickness. Based on the analysis of 100 Rh nanosheets from different regions of the embedded sample, the average thickness of the individual Rh nanosheets is 0.9 ± 0.4 nm. With the help of spherical aberration‐corrected TEM, it can clearly be seen that the as‐prepared Rh nanosheets consist of three to five atomic layers (Figures 1e and S2, Supporting Information). Moreover, it can be observed that the stacking sequence projected along the [111] axis is ABCAB in good agreement with the fcc structure.

The facile synthesis of uniform ultrathin Rh nanosheets in the presence of CO stimulated us to gain a better understanding of their formation mechanism. We believe the formation of ultrathin Rh nanosheets undergoes an attachment mechanism based on the time‐dependent morphology evolution for Rh nanosheets as shown in **Figure** **2**a–d. In the early stage (referring to the temperature just reached at 150 °C), only small particles with a size of 1–2 nm were found randomly dispersed. Electrospray ionization‐mass spectrometry (ESI‐MS) was employed to figure out the nature of these tiny particles. Abundant Rh carbonyl clusters [e.g., Rh_6_(CO)*_x_*, Rh_9_(CO)*_x_*] were clearly identified from the reaction (Figure S3, Supporting Information), consistent with the TEM results. As the reaction proceeded, these clusters tended to aggregate together to form the primal shape of Rh nanosheets and the boundaries started to appear as shown in Figure 2b. Then, Rh nanosheets with typical morphology and long‐edge length of about 120 nm started to form, while there were still many Rh carbonyl clusters left intact. These results suggested that the formation of Rh nanosheets involved the formation of small Rh carbonyl clusters at the early stage, followed by their aggregate formation into nanosheets to minimize their surface energy. With the increased reaction time, the size of Rh nanosheets increased as those small Rh carbonyl clusters continued to attach on the preformed nanosheets and finally ripened into large single crystalline nanosheets.

**Figure 2:**
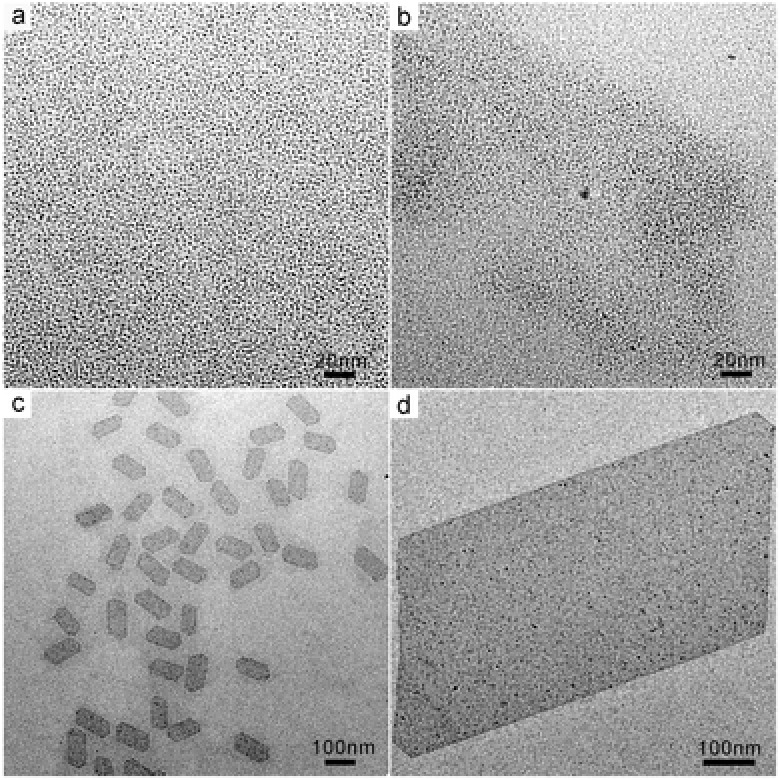
TEM images showing the time‐dependent morphology evolution of the Rh nanosheets formation process at different stages: a) just reached at 150 °C; b) maintained at 150 °C for 0.5 h; c) maintained at 150 °C for 1 h; d) maintained at 150 °C for 3 h.

As has been previously demonstrated by our group, CO serves as the key to the anisotropic growth of the ultrathin Pd nanosheets, while PVP acts as an effective stabilizer to prevent the nanosheets from agglomeration. This is also true in the case of Rh nanosheets. When N_2_ is used instead of CO during the synthesis, the results of time‐track experiments by TEM show that only rhodium particles with nonuniform size and shape were obtained. Neither small clusters nor the subsequent attachment occurred during the reaction process (Figure S4a,b, Supporting Information). The products obtained in the presence of CO but the absence of PVP were generally the aggregates of Rh nanosheets (Figure S4c,d, Supporting Information), whose size and shape were not as uniform as those prepared in the co‐presence of PVP and CO These results confirmed the critical role of CO in the formation of ultrathin nanostructure, involving the formation of small Rh carbonyl clusters followed by their aggregating assembly.

The strong adsorption of CO on Rh played an important role in the anisotropic growth of Rh nanosheets. The CO stripping voltammogram of the freshly prepared Rh nanosheets displayed a major CO‐stripping peak at 0.55 V versus SCE, suggesting that CO mainly adsorbed on Rh(111) (Figure S5, Supporting Information). Both bridge and linear configuration of CO adsorption on the surface of the Rh nanosheets were revealed by Fourier transform infrared (FTIR) studies (Figure S6, Supporting Information). Based on these results, we believe the strong CO adsorption on the basal (111) planes of Rh nanosheets largely prevents the further deposition of Rh carbonyl clusters on (111) and thus the continuing growth of the nanosheets along the [111] direction. The confined growth of Rh nanosheets in the presence of CO is very similar to the situation of Pd nanosheets, indicating that Rh and Pd have similar interactions with CO.

While inducing the anisotropic growth, CO also controlled diameter size of the as‐prepared Rh nanosheets when different‐pressure CO was charged to the reaction system. By tuning the CO pressure in the range of 0.5–2.0 atm, freestanding Rh nanosheets with their long‐edge length ranging from 500 to 1300 nm were readily prepared. As shown in **Figure** **3**, when the applied CO pressures were 0.5, 1.0, 1.5, and 2.0 atm, the average long edges of the obtained Rh nanosheets were 500, 880, 1000, and 1300 nm, respectively. It should, however, be noted that the different‐pressured CO did not vary the ratio of long edge to short edge of the different‐sized Rh nanosheets. The ratio was kept almost constant for all of the obtained Rh nanosheets.

**Figure 3:**
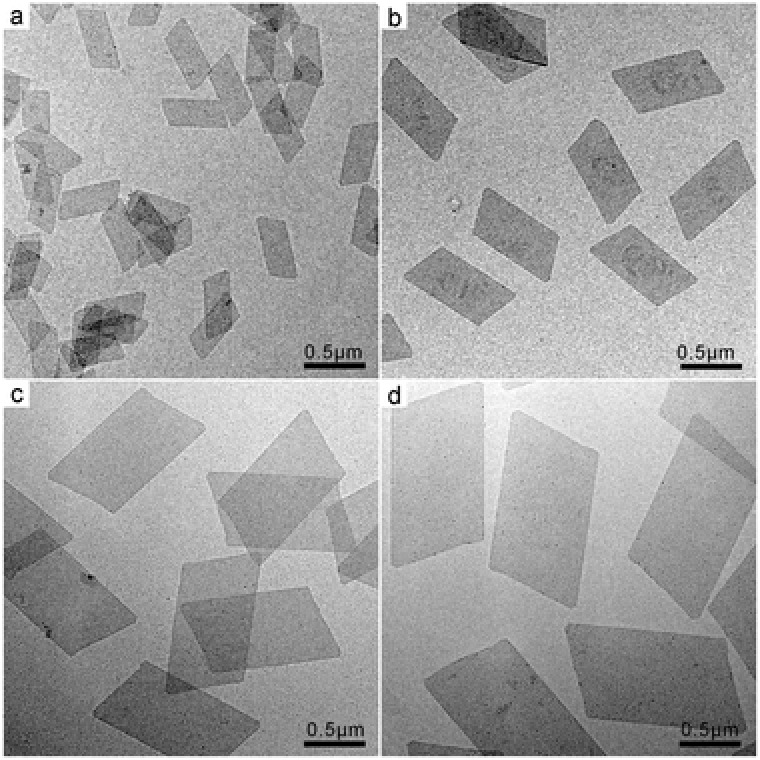
TEM images of Rh nanosheets with tunable size obtained under different CO pressures: a) 0.5 atm CO; b) 1.0 atm CO; c) 1.5 atm CO; d) 2.0 atm CO.

Although it was revealed by the cross‐section TEM studies that Rh nanosheets had a thickness of ≈1 nm, the top‐view TEM images did not seem to support the ultrathin nature of the Rh nanosheets. High‐dark contrasts were associated with the Rh nanosheets, suggesting that the nanosheets should have a greater thickness than 1 nm. We thus applied atomic force microscopy (AFM) to measure the thickness of the Rh nanosheets. As shown in Figure S7 (Supporting Information), the typical thickness of nanosheets ranged from 7.5 to 11.0 nm, indeed much thicker than that observed in the cross‐section TEM images of Rh nanosheets. Such an increment could be simply explained by the capping agent adsorbed on the nanosheets. Together with the observation that many ultrathin Rh nanosheets were stacked together with two to five layers in the cross‐section TEM images of Rh nanosheets (Figure S8, Supporting Information), we deduced that the high‐dark contrasts observed in the top‐view TEM images might be due to that the as‐prepared Rh nanosheets were already in the form of stacked nanosheets. Every Rh nanosheet observed in the top‐view TEM images actually contained two to five ultrathin nanosheets each of which had an individual thickness of ≈1 nm. To further prove this speculation, we loaded the Rh nanosheets on carbon nanotubes to directly observe the side view of Rh nanosheets. According to the analysis of more than 100 TEM images taken from different regions of the sample, each Rh nanosheet was indeed consisting of two to five stacked ultrathin nanosheets (Figure S9, Supporting Information) with a uniform gap distance of ≈0.5 nm between nanosheets.

Many recent studies reported the significant effects of the surfactants (e.g., organic ligands, polymers) on the catalysis of metal nanocrystals. Some believed that surfactant has a negative impact on the catalysis because the catalytic active sites of the nanocrystals could be blocked by capping agents.^22^ However, some also reported that the surfactants can significantly improve the catalysis due to the electronic and steric contributions of surfactants on the nanoparticles.^36^, ^37^ As both surfactant‐capped and surfactant‐free Rh nanosheets were successfully obtained, the synthetic strategy developed in this work provided us with a great opportunity to study the effect of surfactants on the catalysis by ultrathin Rh nanosheets. Because of its wide potential applications in industrial manufacturing processes, e.g., the industrial demands for cyclohexane and the production of low‐aromatic diesel fuels,^38^, ^39^, ^40^ the selective hydrogenation of polycyclic aromatic hydrocarbons was chosen to study the surfactant effect. There are mainly four products from the hydrogenation of anthracene with the hydrogenation of one side ring (A), two side rings (B), the central ring and one side ring (C), or the central ring (D). As clearly shown in **Figure** **4**, the surfactant‐free Rh nanosheets displayed a much better catalytic performance than thesurfactant‐capped ones. It took only 6 h for surfactant‐ free Rh nanosheets to completely convert anthracene, while it took 13 h for the PVP‐capped nanocatalysts under the same conditions. At the early stage of hydrogenation of anthracene (Table S1, Supporting Information), the product with one side ring hydrogenated (A) was the main product. For the surfactant‐capped Rh nanosheets, the selectivity for product A was 100.0% (Table S1, Entry 1, Supporting Information), while in the case of surfactant‐free Rh nanosheets the selectivity was 79.7% (Table S1, Entry 7, Supporting Information), which was the highest during the catalytic process. As the reaction proceeded, products with two saturated side rings and products with a central saturated ring gradually appeared but the selectivity toward products with both a saturated central ring and a side ring was still low. The surfactant‐free Rh nanosheets enjoyed better catalytic activity, while revealing similar products selectivity toward the hydrogenation of anthracene compared with the surfactant‐capped Rh nanosheets. These catalytic results indicated that surfactants or capping ligands used in synthesis can largely decrease the catalytic activity of the obtained metal nanocrystal catalysts.

**Figure 4:**
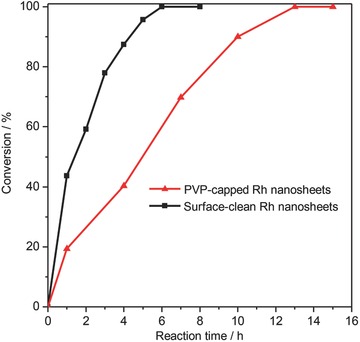
Comparison of surface‐clean and PVP‐capped Rh nanosheets in the catalytic hydrogenation of anthracene. Conditions for the catalysis: Rh nanosheets (1.00 mg), anthracene (178 mg, 1.00 mmol), H_2_ pressure (6 atm), room temperature.

In conclusion, we have demonstrated an effective CO‐confined growth strategy to prepare single‐crystalline Rh nanosheets with atomic thickness and controllable size. Detailed mechanism studies revealed that the formation of Rh nanosheets involved the formation of small Rh carbonyl clusters at the early stage, followed by their aggregation assembly into nanosheets to minimize their surface energy. By understanding the formation mechanism, surfactant‐free Rh nanosheets were also prepared by the same strategy and were revealed to exhibit a far better catalytic activity than surfactant‐capped Rh nanosheets in the hydrogenation of anthracene. Surfactant‐free Rh nanosheets provide us with an opportunity to study the effects of surfactant or capping agent on the catalysis of rhodium nanocrystals. Moreover, the synthetic strategy developed in this work should be applicable to fabricate other ultrathin 2D metal nanosheets with unique physical and chemical properties.

## Experimental Section

*Synthesis of Rhodium Nanosheets with Different Size*: 28.0 mg C_6_H_9_O_6_Rh and 110.0 mg of PVP were dissolved in 10 mL of DMF. The resulting homogenous blue solution was then transferred to a glass pressure vessel. After being charged with CO to 1.0 atm, the vessel was heated from room temperature to 150 °C with a heating rate of 1 °C min^−1^ and maintained at this temperature for 3.0 h under magnetic stirring. After it was cooled down to room temperature, the resulting products were precipitated by acetone, separated through centrifugation, and washed several times with mixture of ethanol and acetone.

*For Size Control*: Different pressure of CO was charged into the reaction system, while keeping other experimental conditions constant. Rh nanosheets with different long‐edge length of 500, 880, 1050, and 1300 nm were obtained by tuning the pressure of charged CO from 0.5, 1.0, 1.5 to 2.0 atm.

*Synthesis of Surface‐Clean Rhodium Nanosheets*: For the synthesis of surface‐clean rhodium nanosheets, no capping agent PVP was added while keeping other experimental conditions the same.

*Preparation of Cross‐Sectional Rhodium Nanosheets Samples for TEM Characterizations*: 0.25 mL of the original dispersion of the as‐prepared rhodium nanosheets was separated and washed three times. After being dried in the vacuum oven, the resulting sample was then embedded in SPI‐PON812 resin and polymerized at 60 °C for 24 h. Sections (50 nm thick) were sliced on a Leica UC 6 microtome equipped with a diamond knife. The cross sectional images of the samples were observed by both TECNAI F‐30 HRTEM and JEOL ARM‐200F double aberration‐corrected scanning transmission electron microscopy.

*General Procedure for Hydrogenation of Anthracene*: In a 50 mL stainless‐steel high pressure autoclave, 5 mL ethanolic dispersion of purified Rh nanosheets or surface clean Rh nanosheets (1.0 mg, determined by ICP analysis), anthracene (178.0 mg, 1.0 mmol), internal standard cyclohexanol (100.0 mg, 1.0 mmol), and 15 mL hexane were added and the reaction mixture was flushed with H_2_ gas for 1 min to replace the air. Then, the outlet valve was closed to maintain 6 atm of H_2_ pressure in the system. The reaction mixture was stirred at room temperature. After the catalytic reaction, the catalyst was separated by centrifugation and the products of hydrogenation of anthracene were analyzed by gas chromatography/mass spectrometry.

*CO Stripping Voltammetry Measurements*: Rh‐nanosheet‐modified working electrodes were fabricated by depositing freshly obtained rhodium nanosheets on a glassy carbon electrode followed by drying in the IR lamp. A platinum foil and a saturated calomel electrode (SCE) were used as the counter electrode and reference, respectively. CO gas (99.99%) was bubbled for 15 min into a 0.1 m HClO_4_ solution with the electrode immersed. The electrode was then quickly transferred to a fresh HClO_4_ solution and the CO stripping voltammetry was recorded at a sweep rate of 5 mV s^−1^.

## Supporting information

As a service to our authors and readers, this journal provides supporting information supplied by the authors. Such materials are peer reviewed and may be re‐organized for online delivery, but are not copy‐edited or typeset. Technical support issues arising from supporting information (other than missing files) should be addressed to the authors.

Supplementary
