# Supplementary material for: Single‐Crystalline Rhodium Nanosheets with Atomic Thickness
Source: Adv Sci (Weinh). 2015 May 8;2(6):1500100. doi: 10.1002/advs.201500100 (PMC5115410; doi:10.1002/advs.201500100)
Supplement: Supplementary file 1 — Supplementary [file ADVS-2-0c-s001.pdf]

Copyright WILEY-VCH Verlag GmbH & Co. KGaA, 69469 Weinheim, Germany, 2015.

ADVANCED SCIENCE
Open Access

Supporting Information

for *Adv. Sci.*, DOI: 10.1002/advs.201500100

Single-Crystalline Rhodium Nanosheets with Atomic Thickness

*Li Zhao, Chaofa Xu, Haifeng Su, Jinghong Liang, Shuichao Lin, Lin Gu, Xingli Wang, Mei Chen, and Nanfeng Zheng**

Copyright WILEY-VCH Verlag GmbH & Co. KGaA, 69469 Weinheim, Germany, 2013.

Supporting Information

Single-Crystalline Rhodium Nanosheets with Atomic Thickness

Li Zhao,[a] Chaofa Xu,[a] Haifeng Su,[a] Jinghong Liang,[a] Lin Gu,[b] Xingli Wang,[a] Mei Chen,[a] and Nanfeng Zheng[a]*

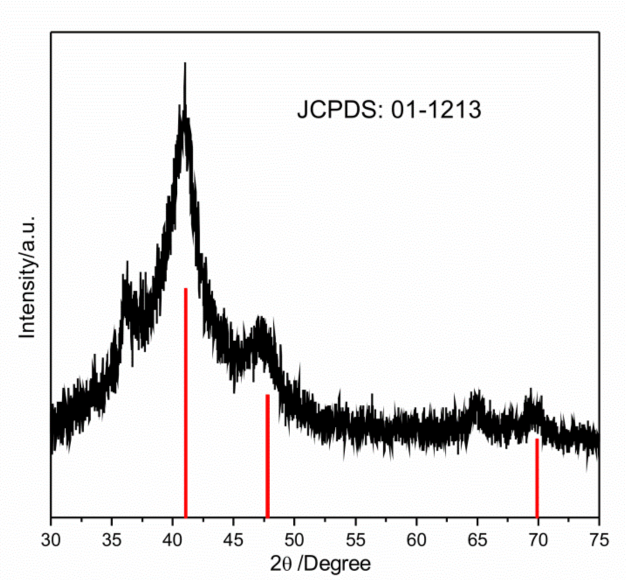

Figure S1. X-ray powder diffraction (XRD) pattern of the as-prepared rhodium nanosheets, which can be indexed to a face-centered cubic phase of rhodium (JCPDS: 01-1213).

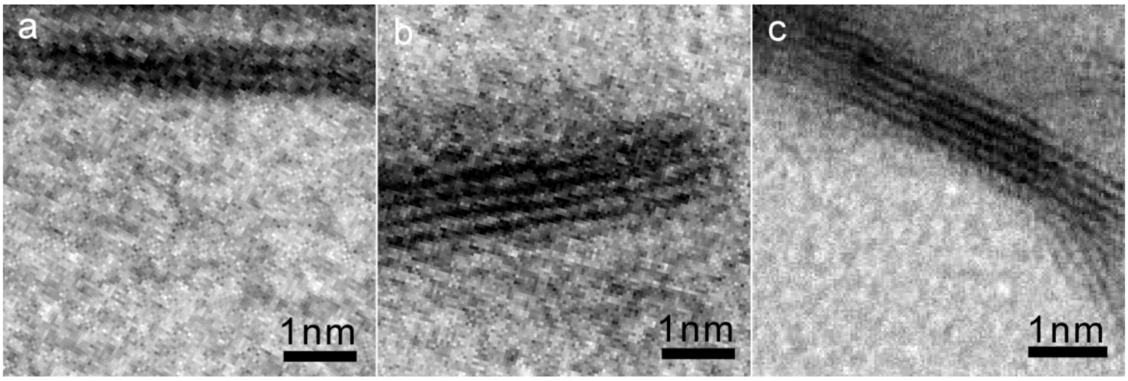

Figure S2. High resolution bright-field images of the cross-sectional single rhodium nanosheets with 3-5 atomic layers

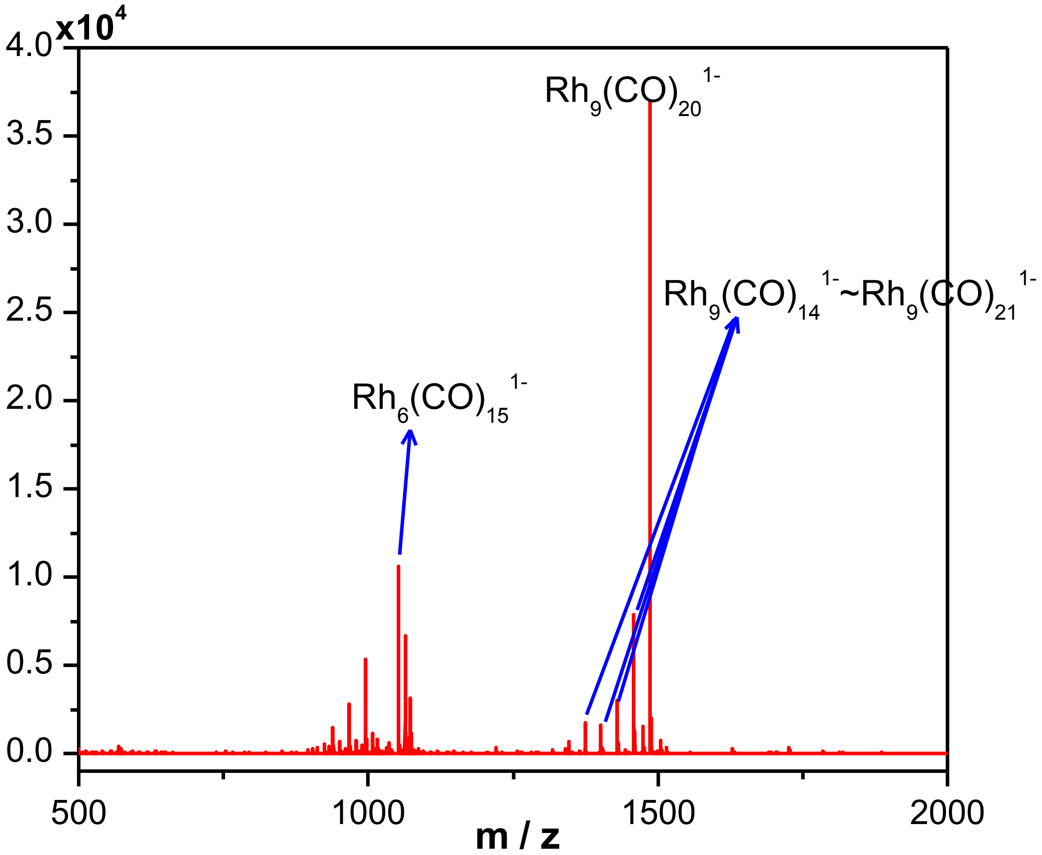

Figure S3. Mass spectra of the reaction solution that just reached 150 ℃ during the heating process in the synthesis of Rh nanosheets. This indicates that the rhodium carbonyl clusters were formed at this period of time.

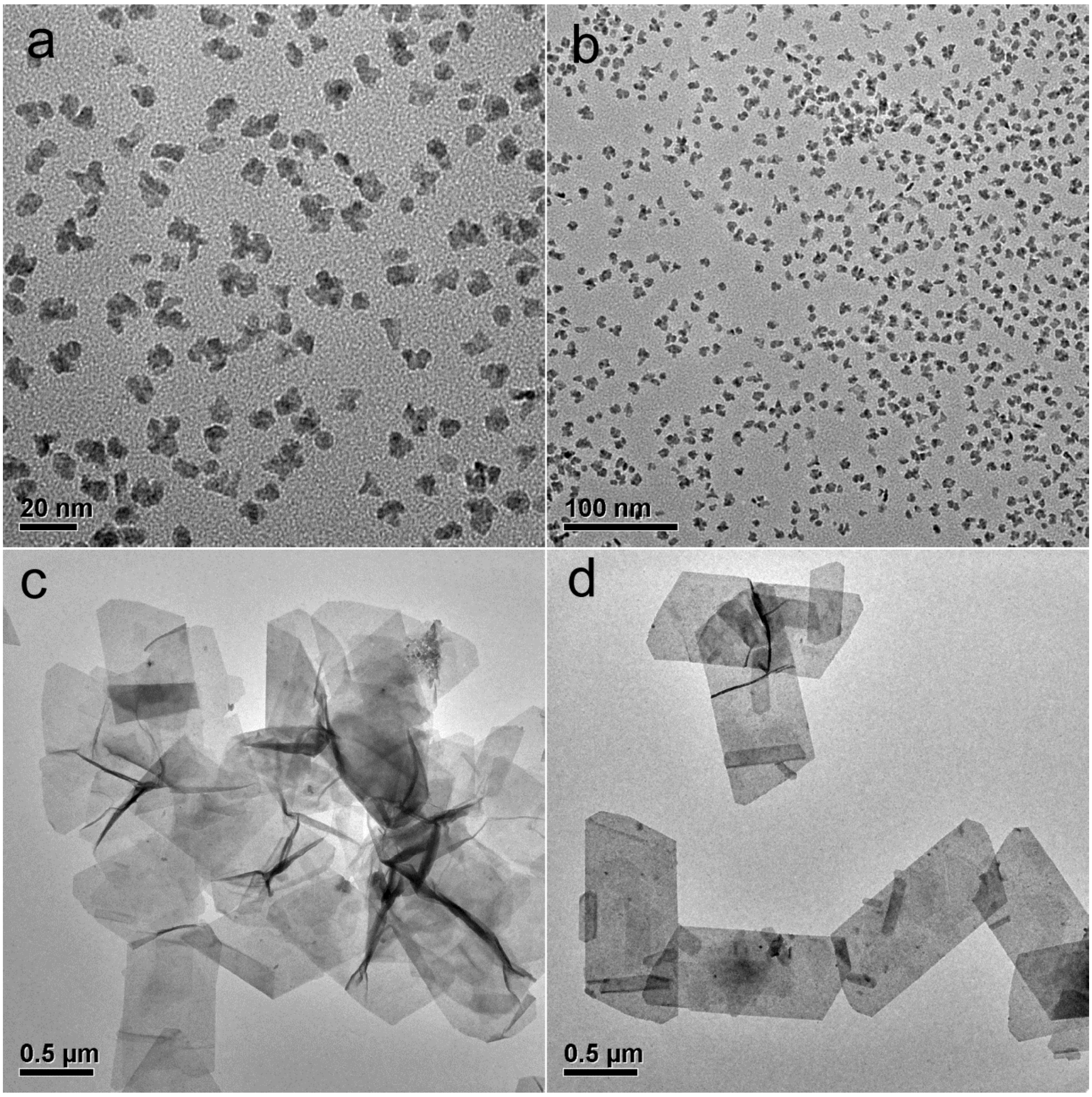

Figure S4. Representative TEM images obtained from the contrast experiments under different conditions: (a-b) conducted under N_2 atmosphere instead of CO while keeping other experimental condition constant. (c-d) conducted in absence of PVP.

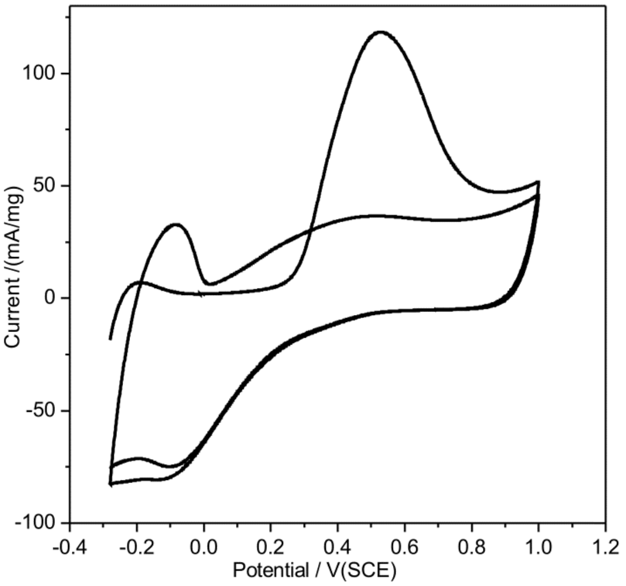

Figure S5. CO stripping voltammetry of rhodium nanosheets obtained in 0.1 M $HClO_4$ solution under 5 mV/s scan rate. The peak at 0.55 V observed in CO stripping curve can be assigned to the CO adsorption on the (111) facets of the as-prepared Rh nanosheets. Figure.S5 is attributed to Rh (111). (T. H. M. Housmans, J. M. Feliu, M. T. M. Koper, *J Electroanal Chem* **2004**, *572*, 79-91.)

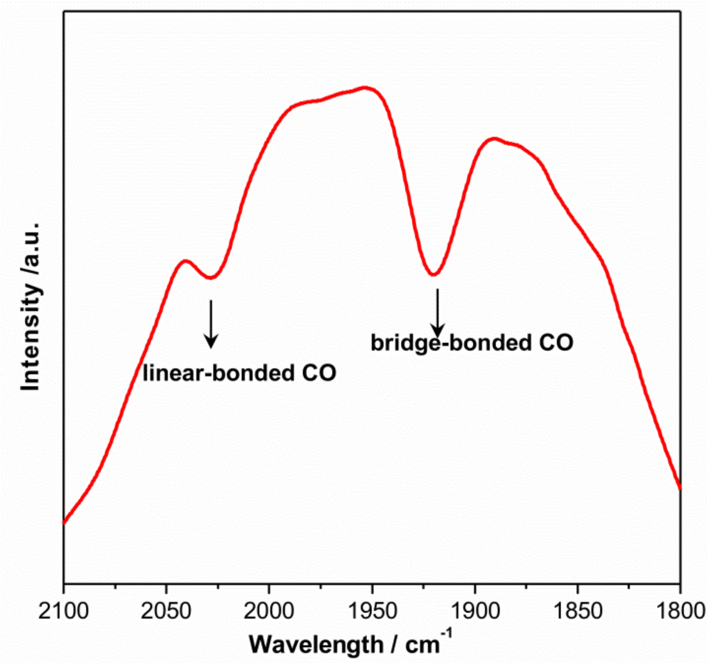

Figure S6. FTIR spectra of the freshly prepared rhodium nanosheets dispersed in DMF solvent without introducing extra CO after synthesis indicating CO adsorption on rhodium nanosheets.

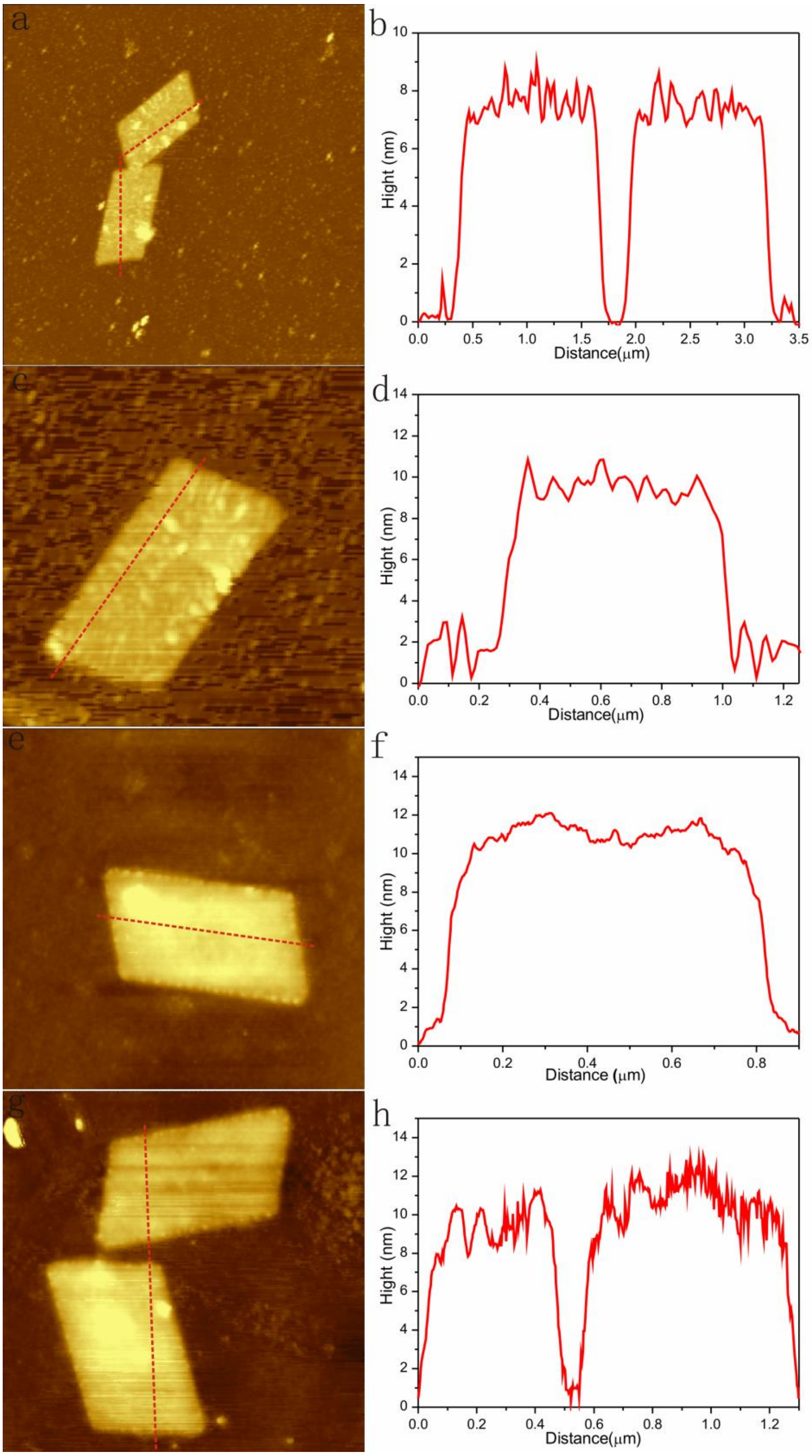

Figure S7. Representative AFM images of the rhodium nanosheets for the thickness measurements. (a, c, e, g) AFM images of typical rhodium nanosheets obtained in the presence of PVP. (b, d, f, h) Height profiles along the dashed line in their respective left AFM images. All the nanosheets were deposited on Si wafers for AFM analysis.

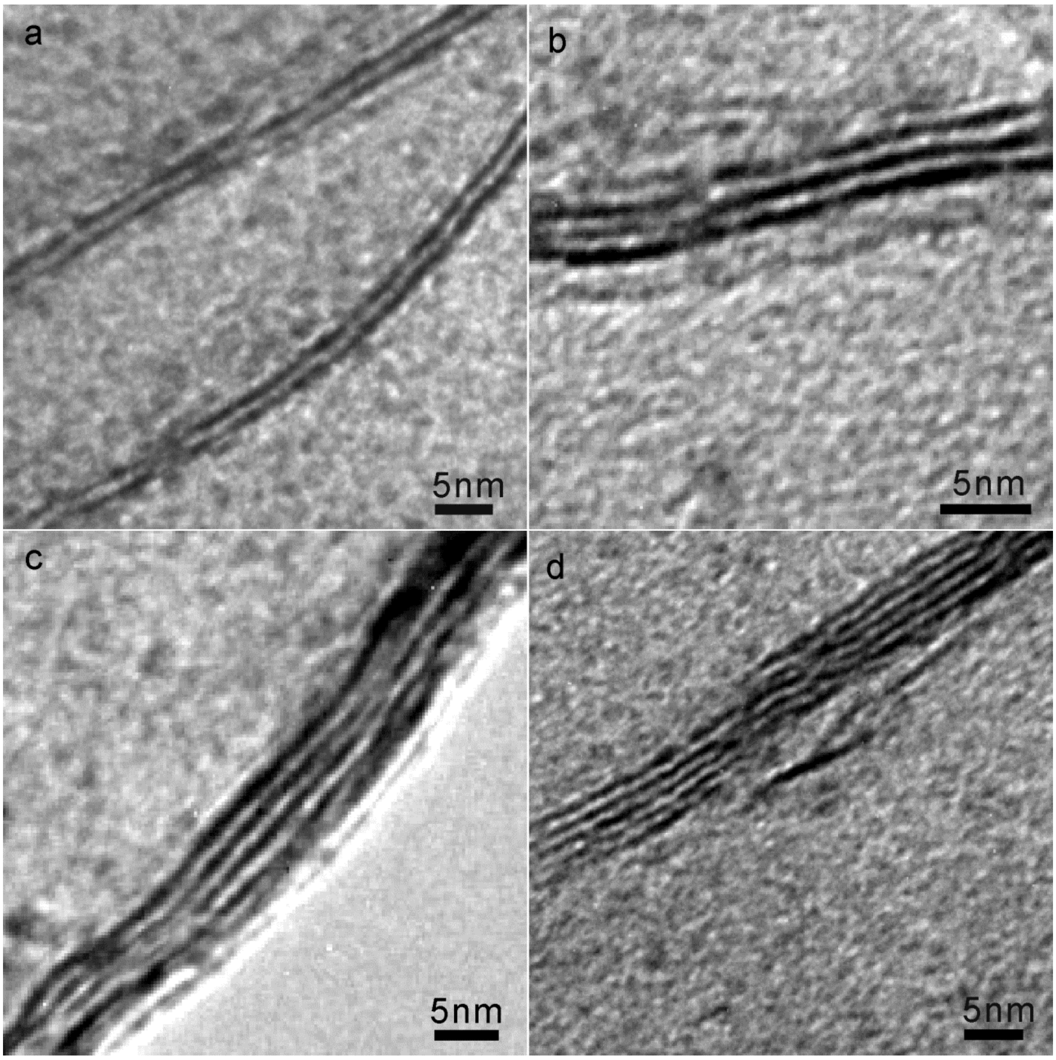

Figure S8. TEM images of the cross-sectional rhodium nanosheets obtained by microtome, which provide evidence for the lamer structure of rhodium nanosheets and each unit contains 2-5 ultrathin rhodium nanosheets.

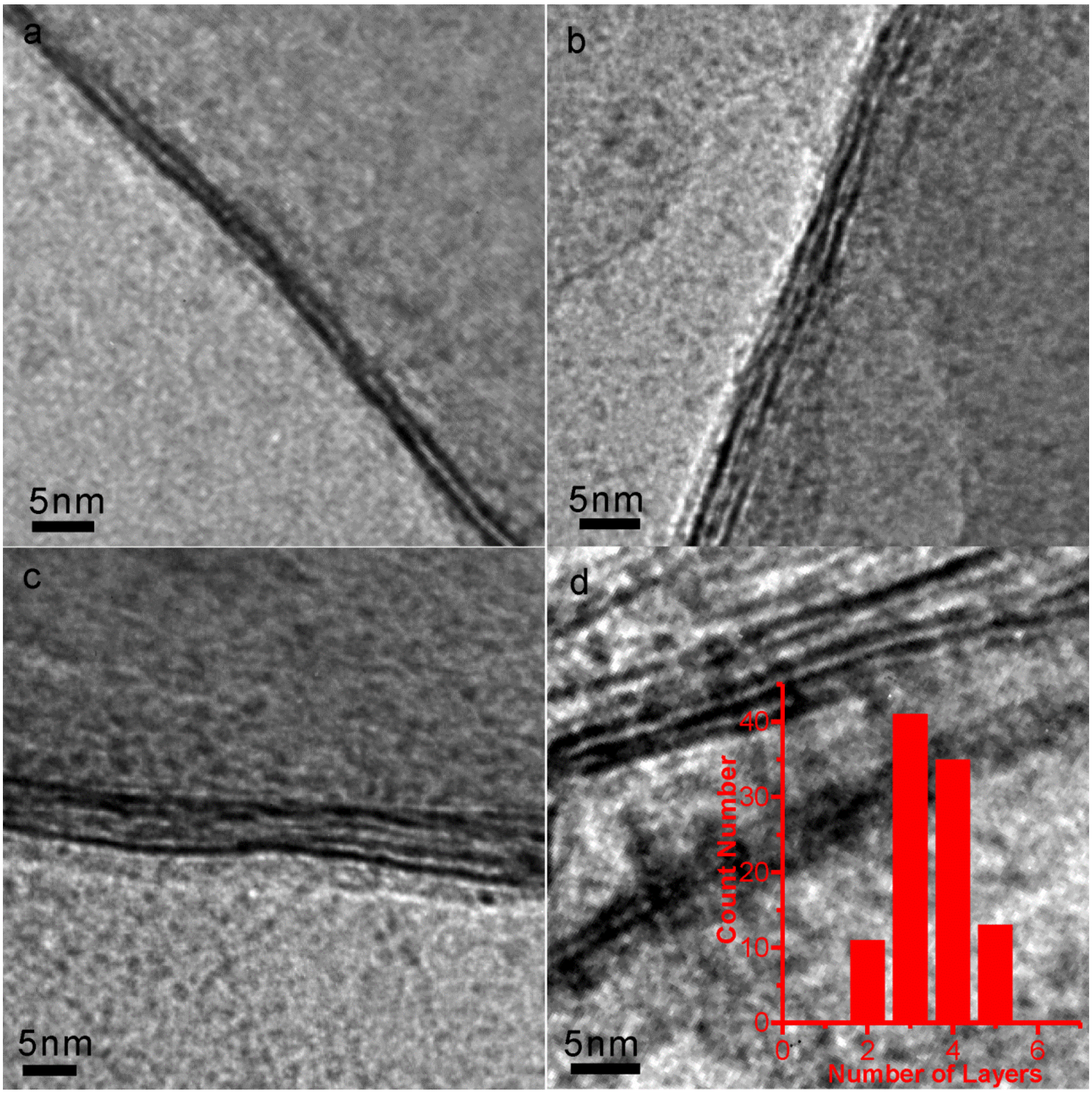

Figure S9. TEM images of rhodium nanosheets loaded on carbon nanotubes, which confirm the lamer structure. The distribution of different numbers of ultrathin layers is shown in the inset, which was obtained from the analysis of 100 TEM images taken from different regions of the sample.

Table S1. Comparison of catalytic activities and selectivities of Rh nanosheets for the hydrogenation of anthracene.[a]

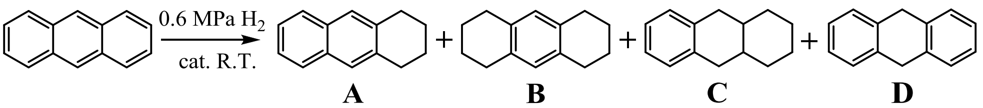

Entry	Cat.[b]	P [atm]	t [h]	Conv.[c] [%]	Selectivity [%] A	B	C	D
1	Rh-a	6.0	1.0	19.4	100.0	ND	ND	ND
2	Rh-a	6.0	4.0	40.3	76.9	14.8	2.5	5.8
3	Rh-a	6.0	7.0	69.8	76.6	18.1	3.0	2.3
4	Rh-a	6.0	10.0	90.1	62.2	18.8	4.6	14.4
5	Rh-a	6.0	13.0	100.0	60.7	17.3	5.9	16.1
6	Rh-a	6.0	15.0	100.0	50.4	21.0	7.3	21.3
7	Rh-b	6.0	1.0	43.7	80.7	12.7	ND	6.6
8	Rh-b	6.0	2.0	59.1	76.2	12.9	ND	10.9
9	Rh-b	6.0	3.0	77.9	77.9	11.9	ND	10.2
10	Rh-b	6.0	4.0	87.4	70.2	11.0	4.0	14.8
11	Rh-b	6.0	5.0	95.7	69.7	13.8	3.7	12.8
12	Rh-b	6.0	6.0	100.0	68.9	16.6	3.4	11.1
13	Rh-b	6.0	8.0	100.0	61.3	19.5	3.7	15.5

[a] Reaction conditions: 1.0 mmol of anthracene and 1.0 mmol% of Rh nanosheets (based on ICP results) in a mixture of hexane and ethanol at room temperature. [b] PVP-capped rhodium nanosheets (Rh-a), surface-clean rhodium nanosheets (Rh-b). [c] Conversion determined by GC-MS.
